# Anemia and undernutrition in intestinally parasitized schoolchildren from Gakenke district, Northern Province of Rwanda

**DOI:** 10.1371/journal.pone.0262361

**Published:** 2022-01-06

**Authors:** María José Irisarri-Gutiérrez, Lucrecia Acosta, Lucy Anne Parker, Rafael Toledo, Fernando Jorge Bornay-Llinares, José Guillermo Esteban, Carla Muñoz-Antolí

**Affiliations:** 1 Área de Parasitología, Departamento Farmacia y Tecnología Farmacéutica y Parasitología, Facultat de Farmàcia, Universitat de València, Valencia, Spain; 2 Área de Parasitología, Departamento Agroquímica y Medioambiente, Facultad de Farmacia, Universidad Miguel Hernández de Elche, Alicante, Spain; 3 Laboratorio de Análisis Clínicos, Sanatorio “Fontilles”, Vall de Laguar, Alicante, Spain; 4 Departamento Salud Pública, Historia de la Ciencia y Ginecología, Universidad Miguel Hernández de Elche, Alicante, Spain; 5 Centro de Investigación Biomédica en Red (CIBER) Epidemiología y Salud Pública, Madrid, Spain; University of Western Australia, AUSTRALIA

## Abstract

**Background:**

Rwanda is a sub-Saharan country, where intestinal parasite infections, anemia and undernutrition coexist. The purpose of this research is to study the relationship between intestinal parasite infections and undernutrition/anemia to clarify the priorities of intervention in the rural area of Gakenke district in the Northern Province of Rwanda.

**Materials and methods:**

A total of 674 students from Nemba I School, participated in a cross-sectional study, in which their parasitological and nutritional status were analysed. Statistical analysis was performed by χ2 test, univariate analysis and Odds ratios (OR).

**Results:**

A total of 95.3% of children presented intestinal parasitism, most of whom (94.5%) infected by protozoa and 36.1% infected by soil-transmitted helminths (STH), with *Trichuris trichiura* (27.3%) being the most prevalent. Multiple infections were found to be high (83.8%), with protozoa and STH co-infections in 30.6%. STH infections were mainly of low/moderate intensity. Neither infection nor STH infection of any intensity profile, was significantly related to anemia. In addition, STH infection, regardless of the intensity profile, was not associated with stunting, underweight or thinness. There was no difference between genders nor among ages in odds of anemia and nutritional status in STH-infected schoolchildren.

**Conclusion:**

Multiparasitism remains high among Rwandan schoolchildren and is likely to cause nutritional problems. This work emphasizes the importance of keeping up health programs to reduce the prevalence of infection.

## Introduction

According to the Food and Agriculture Organization of the United Nations, the majority of undernourished individuals (mainly involving stunting, underweight and thinness) live in sub-Saharan Africa, Southern Asia and the Caribbean [1]. The problem is particularly severe in Africa where 20.2–48.1% of children are stunted and 14–36.5% are underweight [2, 3].

School-age children are the group most severely affected by intestinal parasites, suffering the greatest morbidity caused by these parasites [4]. Moreover, multiparasite infections at various intensities might have different effects on the undernutrition and anemia status of children [5], with chronic intestinal protozoa infections being one of the most recognized causes [4].

In the tropics and subtropics, where soil-transmitted helminth (STH) infections are common parasitic infections [5], several studies have found associations between these infections and child malnutrition through a subtle reduction in digestion and absorption, chronic inflammation and loss of nutrients, leading to stunting, underweight and thinness [3, 6–9]. Additionally, an individual harboring STHs might experience anemia and gastrointestinal physiological damage able to exacerbate nutritional deficiencies [10, 11]. The link between Hookworm and anemia is well known [12, 13], but there are different opinions about other STH infections able to, directly or indirectly, affect the hemoglobin (Hb) level leading to anemia [5, 14–16]. However, heavy intensities of STH infection have also been associated to anemia [17].

Rwanda is a small landlocked country in Central Africa, bordering Uganda to the North, Burundi to the South, Democratic Republic of the Congo to the West, and Tanzania to the East. In this country, parasite infections, anemia and undernutrition coexist. Anemia remains a public health problem in Rwanda but further investigation is needed into the causal origin of anemia [18]. National surveys have shown a wide distribution of STH infections in Rwanda with significant variations between districts [19–21]. Mupfasoni [17] determined 21.2% of multiparasite infections in two districts (Musanze and Burera) of the Northern Province in Rwanda to back up deworming programs, and Geus [22] analyzed co-infections with *Giardia*, *Ascaris* and *Plasmodium* up to 24% in Huye district in the Southern Province, relating them to hemoglobin, anemia and anthropometric data in Rwandan children aged 6–10 years. Apart from these studies, there are no other studies that analyzed the relationship of parasitic infections with anemia and nutritional morbidities in other areas of Rwanda.

The aim of this work is to study the relationship between intestinal parasite infections and undernutrition/anemia to clarify the priorities of intervention in a rural area of Northern Province of Rwanda.

## Materials and methods

### Study area and population

**Fig 1:**
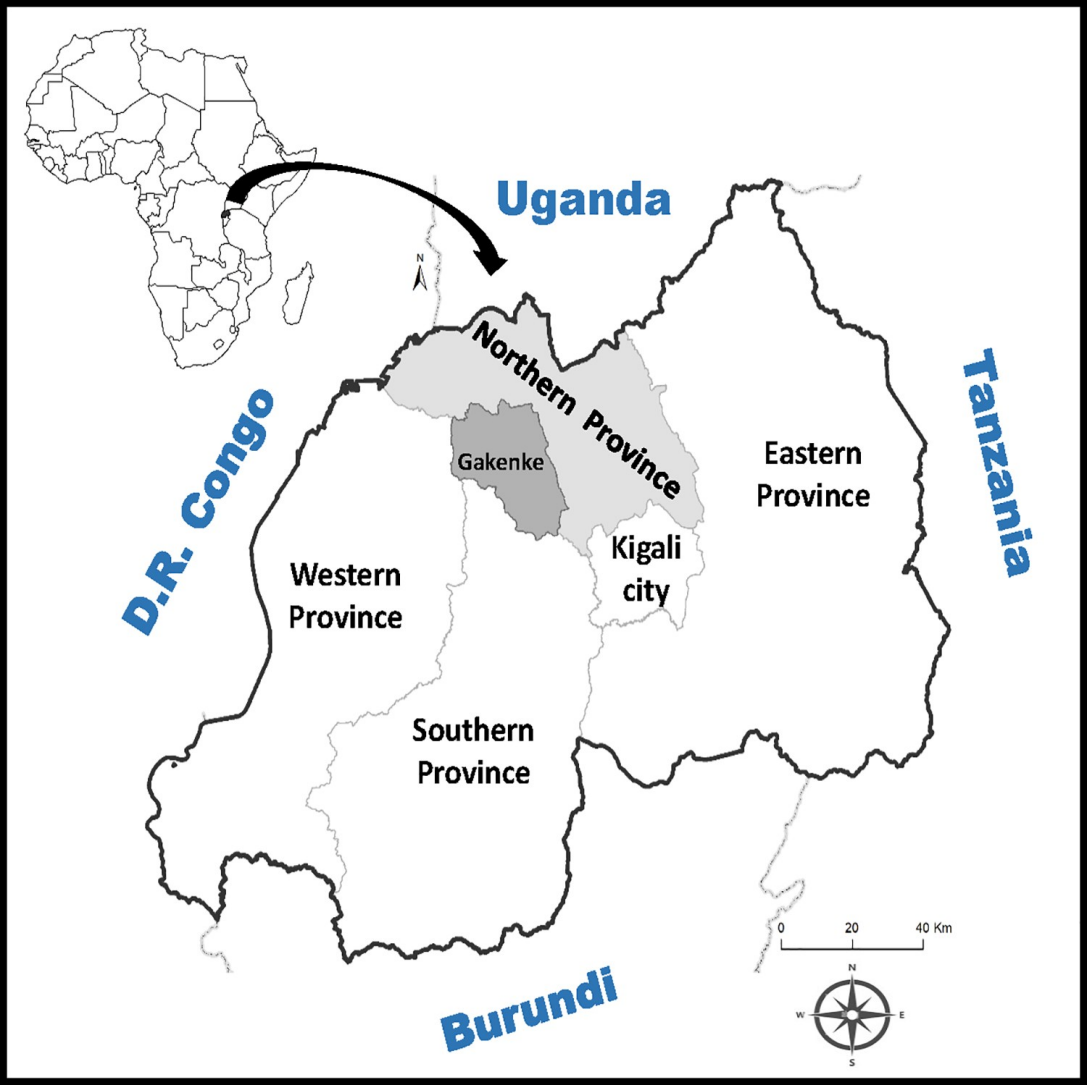
Map of the African continent showing the situation of Rwanda and its different provinces; the location of Gakenke district in Northern Province is highlighted in dark grey. (Administrative limits from GADM v3.6, ArcGIS v.10.6.).

Rwanda is divided in 5 provinces (Northern-, Eastern-, Southern-, and Western Provinces and the city of Kigali), with 5 districts making up the Northern Province. We conducted a cross-sectional study in E.P. Nemba I (École Primaire Nemba I) public school, situated in the Nemba sector (Gakenke district, Northern Province of Rwanda) (Fig 1). This school hosts several volunteer and international cooperation projects from Universidad Miguel Hernández de Elche (Spain). Nemba sector is a mountainous rural area with a population of 15,000 inhabitants; the majority of whom live on subsistence farming and agriculture. Due to the altitude (2,000m), Nemba sector presents a humid climate with an average annual temperate ranging between 16° and 29°C and abundant rainfalls (1,100–1,500 mm per year) [23].

### Study design, procedure and sampling

We used a cross-sectional study design to recruit students during August to September 2011. All 771 schoolchildren attending 2^nd^ to 6^th^ grade primary education at E.P. Nemba I were eligible for inclusion in the study. Students who were absent on the sampling day, and students who did not provide informed consent, were excluded. A total of 674 schoolchildren (87.4%) participated voluntarily in the cross-sectional study, of whom 656 (97%) provided blood samples and were included in the analysis of anemia status.

Two days before recruitment, consent forms, an empty plastic container for stool collection and sampling instructions were distributed to students who attended their classes. At recruitment, basic demographic data (name, sex and age) were recorded and students were weighed (using a previously calibrated mechanical scale) and measured (using a rigid metal tape measure with children positioned according to the Frankfurt vertical plane). Blood samples were taken by Nemba District Hospital laboratory technical staff who performed the blood counts using an automated hematological system. Containers with stool samples were collected and the Kato-Katz slides were initially examined within 1 h of preparation to avoid over clarification of some helminth eggs. The rest of the fecal sample was fixed in 10% formalin solution (1:3) and transported by plane to the Laboratory of Parasitology of University of Valencia (Valencia, Spain) to be analyzed.

Each student´s demographic, anthropometric and anemia status data were recorded into a Microsoft Excel sheet. In this digital version, data was anonymized using numerical codification for each student.

### Parasitological examinations

One stool sample per child was collected. A Kato-Katz slide was made from each stool sample following WHO recommendations, using a template delivering approximately 41.7 mg of feces [24]. All samples were processed by wet mount and by a sedimentation concentration technique [25]. One aliquot of sediment obtained with the concentration technique was stained using a modified Ziehl-Neelsen technique for detection of coccidian protozoa [26].

The sediments of the concentration technique and the Kato-Katz slides were used to obtain the prevalence data. Based on the techniques used, the prevalence obtained for the pinworm *Enterobius vermicularis* may not be considered definitive as anal swabs would be the adequate technique for the detection of the eggs of this nematode species. However, this technique could not be used given its methodological difficulties in field work. The Kato-Katz slides were analyzed for egg counts, and the infection intensities were subdivided into light, moderate and heavy egg excretion intensities according to thresholds proposed by WHO [27]: for *T*. *trichiura*, they were 1–999 epg, 1,000–9,999 epg and ≥10,000 epg; for *A*. *lumbricoides*, they were 1–4,999 epg, 5,000–49,999 epg and ≥50,000 epg; and for hookworm, they were 1–1,999 epg, 2,000–3,999 epg and ≥4,000 epg.

### Anemia status

Anemia status, age groups and hemoglobin levels adjustment were applied for each schoolchild, as described by Sullivan [28], since the district has variations in altitude at sea level (between 1,700 and 2,700 meters). Students could be classified with presence or absence of anemia, using the hemoglobin cut-off values (g/dL) [29] according to age and gender. In this study, the cut-off value of 11.5 g/dL was used for schoolchildren between 5 and 12 years; the value of 12 g/dL for schoolchildren aged between 12 and 15 years and for girls over 15 years of age; and, finally, the value of 13 g/dL for boys aged 15 and above.

### Anthropometric status

With the data of height, weight and age, Z-scores of anthropometric indicators [height-for-age (HAZ), weight-for-age (WAZ) and Body Mass Index-for-age (BAZ)] were calculated with the AnthroPlus software [30]. WAZ was only calculated for schoolchildren younger than 10 years old.

These indicators define stunting, underweight and thinness as the Z-score of HAZ, WAZ and/or BAZ < -2 SD, respectively. The World Health Organization reference population [31] was used to compare the nutritional results of the study population.

### Study variables and statistical analysis

Statistical analysis was performed with the STATA 12 software (StataCorp, U.S.A.). Descriptive analysis was executed by calculating absolute and relative frequency and prevalences, together with 95% confidence intervals (95% CI). We performed the χ2 test and calculated Odds ratios (OR) to assess associations. Depending on the different analyses performed the independent variables were parasitic infection (any infection, type of infection and STH intensity as Light+Moderate/Heavy), nutritional status (presence/absence stunting, underweight and thinness) and anemia status. The main dependant variables were gender and age-group. To assess the relationship between parasitic infection and anemia or nutritional status, the presence and characteristics of the parasitic infection was considered as a dependant variable. Observed differences were considered significant, if the *P* value≤ 0.05.

### Ethics statements

Only voluntary schoolchildren who submitted an informed consent signed by their parents or guardians participated in the study. The Mayor of Gakenke district and the Ruhengeri Diocese of Catholic Church gave permission to enter the schools, in agreement with the University Miguel Hernandez de Elche (Spain) study (Exp. 3055/2009) involving human participants. The research protocol was approved by “Experimental Research Commission on Ethics” from University Miguel Hernandez de Elche (Spain) (Ref.:DF-MPA-001-11) and University of Valencia (Spain) (Ref.:H1474970118385).

## Results

### Demographic characteristics, anemia and nutritional status

A total of 674 students (330 boys, 344 girls) being between 6 and 18 years of age (mean ± SD = 11.00 ± 2.33 years old) formed the study population. The prevalence of anemia (26 children, 4%) was low. Anemic children were more frequently girls and members of the youngest age group (5–12 years old) (57.7% and 61.5%, respectively). No significant differences were detected by gender and age groups (S1 Table).

The most prevalent nutritional morbidity was stunting (32.8%), followed by underweight (3.7%) and thinness (2.1%) (Table 1). Of schoolchildren affected by stunting, 57.4% were males and they were more frequently stunted compared to females (38% of males compared to 27% of females, p = 0.018). Similarly, stunting varied by age group with significantly higher prevalence of stunting among children aged 14–18 years (49 children, 44.9% of this group, p = 0.0001). Thinness was also significantly increased in older children (14–18 years of age) (p<0.0001).

**Table 1:** Nutritional status by gender and age groups.

		Stunting (HAZ)			Underweight (WAZ)			Thinness (BMI)		
		n (%)	OR (95%CI)	*P* value	n (%)	OR (95%CI)	*P* value	n (%)	OR (95%CI)	*P* value
Total N = 674		221 (32.8)			25 (3.7)			114 (2.1)		
Gender										
	Male	127 (57.4)	1.43 (1.07–1.92)	0.018	11 (44.0)	0.78 (0.34–1.75)	0.686	10 (71.4)	2.52 (0.80–9.31)	0.179
	Female	94 (42.5)			14 (56.0)			4 (28.6)		
Age-group										
	6–10	75 (34.1)	5.71 (3.59–9.40)	0.0001	25 (100)			4 (28.6)		
	11–13	96 (43.6)			0 (0.0)	NA	NA	5 (35.7)	2.90 (2.23–3.77)	<0.0001
	14–18	49 (22.3)			0 (0.0)			5 (35.7)		

Nutritional morbidity prevalence and distribution (%) by gender and age-groups among schoolchildren of Nemba I (Gakenke district, Northern Province, Rwanda). N = number of schoolchildren studied; n = number of schoolchildren with nutritional morbidity; OR = Odd ratios; 95%CI = 95% confidence interval; NA = not available.

### Overall prevalence and intensity of parasite infection

A total of 95.3% schoolchildren presented intestinal parasitism, most of whom (94.5%) were infected by protozoa and 36.1% by helminths. A total of at least ten protozoan species were found, mainly *Blastocystis* sp. in 89.9%, *Entamoeba* complex in 21.8% and *Giardia intestinalis* in 21.1% of the schoolchildren, while at least seven helminth species were found, with *Trichuris trichiura* (27.3%) being the most prevalent, followed by *Ascaris lumbricoides* (10.8%) (Table 2).

**Table 2:** Protozoa and helminth infections prevalence.

	E.P. Nemba I school		
	N = 674		
	n	%	95%C.I.
Protozoa	637	94.5	92.6–96.1
*Entamoeba coli*	386	57.2	53.5–60.9
*Entamoeba hartmanni*	380	56.4	52.6–60.1
*Entamoeba* complex	147	21.8	18.8–25.1
*Endolimax nana*	614	91.1	88.8–93.1
*Iodamoeba bütschlii*	180	26.7	23.5–30.1
*Giardia intestinalis*	142	21.1	18.1–24.3
*Chilomastix mesnili*	20	2.9	1.9–4.5
*Enteromonas hominis*	2	0.3	0.04–0.9
*Cryptosporidium* sp.	4	0.6	0.2–1.4
*Blastocystis* sp.	606	89.9	87.5–92.0
Helminths	243	36.1	32.5–39.7
*Hymenolepis diminuta*	1	0.1	0.01–0.7
*Hymenolepis nana*	2	0.3	0.04–0.9
*Taenia* sp.	2	0.3	0.04–0.9
*Enterobius vermicularis*	1	0.1	0.01–0.7
*Trichuris trichiura*	184	27.3	23.8–30.5
*Ascaris lumbricoides*	73	10.8	8.4–13.1
Hookworm	4	0.6	0.2–1.4
TOTAL INFECTION	642	95.3	93.4–96.7
NO INFECTION	32	4.7	3.3–6.6

Prevalence (%) of intestinal protozoa and helminth infections among schoolchildren of Nemba I (Gakenke district, Northern Province, Rwanda). N = number of schoolchildren studied; n = number of schoolchildren parasitized; 95%C.I. = 95% confidence interval.

Multiple infection (83.8%) was more prevalent than single infection (16.2%). Three different species show the highest percentage of co-infection (19.2%) with nine species in two schoolchildren being the maximum number of multiparasitism detected. Multiparasitism of STH species only occurred in 1.1% of the infected schoolchildren, and co-infection by protozoa and STH species in 30.6%. The infected schoolchildren were both males and females (p = 0.144), and were frequently schoolchildren aged 6–10 years (p<0.001) (Table 3).

**Table 3:** Infection distribution (%) according to type of infection and demographic characteristics (gender and age-groups).

	Nemba I school			
	N = 642			
	n	%	95%C.I.	*P* value
Type of infection				
Single	104	16.2	13.5–19.2	p<0.001
Multiple	538	83.8	80.8–86.5	
Gender				
Males	311	48.4	44.6–52.3	0.144
Females	331	51.6	47.7–55.4	
Age-groups (years)				
6–10	297	46.3	42.4–50.1	
11–13	267	41.6	37.8–45.4	p<0.001
14–18	78	12.1	9.8–14.9	

Infection distribution (%) according to type of infection and demographic characteristics (gender and age-groups) among schoolchildren of Nemba I (Gakenke district, Northern Province, Rwanda). N = number of infected schoolchildren; n = number of schoolchildren with this infection characteristic; 95%C.I. = 95% confidence interval; *P* value obtained by χ2 test.

STH infections were mainly of low/moderate intensity, with only one student presenting a heavy intensity *A*. *lumbricoides* infection. Males were equally infected as females with low/moderate intensity STH infections, while those in the 6-10-year range resulted statistically more infected with *T*. *trichiura* (p<0.0001) and with *A*. *lumbricoides* (p<0.00001) than those of older age groups (Table 4).

**Table 4:** STH intensity of infection.

		*T*. *trichiura*			*A*. *lumbricoides*			Hookworm		
		N = 184			N = 73			N = 4		
		n (%)			n (%)			n (%)		
		Lig.+Mod.	Heavy	Total (%)	Lig.+Mod.	Heavy	Total (%)	Lig.+Mod.	Heavy	Total (%)
Gender										
	Male	87 (47.3)	0 (0.0)	87 (47.3)	38 (52.1)	0 (0.0)	38 (52.1)	2 (50.0)	0 (0.0)	2 (50.0)
	Female	97 (52.7)	0 (0.0)	97 (52.7)	34 (46.5)	1 (100)	35 (47.9)	2 (50.0)	0 (0.0)	2 (50.0)
Age-groups (years)										
	6–10	93 (50.5)*	0 (0.0)	93 (50.5)	34 (46.5)*	1 (100)	35 (47.9)	1 (25.0)	0 (0.0)	1 (25.0)
	11–13	63 (34.3)	0 (0.0)	63 (34.3)	26 (35.6)	0 (0.0)	26 (35.6)	2 (50.0)	0 (0.0)	2 (50.0)
	14–18	28 (15.2)	0 (0.0)	28 (15.2)	12 (16.4)	0 (0.0)	12 (16.4)	1 (25.0)	0 (0.0)	1 (25.0)

Infection distribution (%) of light, moderate and heavy STH infections, among schoolchildren of Nemba I (Gakenke district, Northern Province, Rwanda), by gender and age groups. N = number of schoolchildren parasitized; n = number of schoolchildren parasitized of each group; Lig.+Mod. = Light + Moderate infections

* = *P* value≤0.05 obtained by χ2 test.

### Association between infection, anemia and nutritional status

The prevalence of anemia was low in the sample. Of the 26 anemic schoolchildren found, 92.3% were infected with at least one parasitic infection, 26.9% of whom had a STH infection. Our results showed no significant association between STH infection and anemia (Table 5). We were unable to consider anemia and helminth species individually (*T*. *trichiura*, *A*. *lumbricoides* and Hookworms), due to the low number of children with anemia.

**Table 5:** Odds ratios for anemia in infected schoolchildren.

				Anemia				
				N = 26				
				n (%)	OR		95%CI	*P* value
any infection				24 (92.3)		0.857	0.22–5.54	0.845
any STH				7 (26.9)		2.266	0.86–5.43	0.120
*T*. *trichura*								
Lig.+Mod.				6 (23.1)		1.212	0.43–2.98	0.877
Heavy				0 (0.0)		NA	-	-
*A*. *lumbricoides*								
	Lig.+Mod.			1 (3.8)		0.680	0.03–3.80	0.948
	Heavy			0 (0.0)	NA		-	-
Hookworm								
	Lig.+Mod.			0 (0.0)	NA		-	-
	Heavy			0 (0.0)	NA		-	-

Anemia distribution (%) in STH infected schoolchildren of Nemba I (Gakenke district, Northern Province, Rwanda). N = number of anemic schoolchildren; n = number of schoolchildren parasitized; Lig.+Mod. = Light + Moderate infections; OR = Odd ratios; 95%CI = 95% confidence interval; NA = not available.

When the nutritional indicators were analyzed, in relation to infection, values appear above 90% (BMI: 100%; WAZ: 96.0%; HAZ: 95%). Among schoolchildren with STH, the frequency of children with stunting, underweight or thinness was between 14.3% and 16.0%, and no significant association between any STH infection intensity was related with any nutritional indicators (Table 6).

**Table 6:** Odds ratios for nutritional status in infected school children.

	Stunting (HAZ)				Underweight (WAZ)				Thinness (BMI)			
	N = 221				N = 25				N = 14			
	n (%)	OR	95%CI	*P* value	n (%)	OR	95%CI	*P* value	n (%)	OR	95%CI	*P* value
any infection	210 (95.0)	1.949	1.00–4.03	0.073	24 (96.0)	2.669	0.48–56.3	0.515	14 (100)	1.55	0.26–33.69	0.992
any STH	33 (14.9)	1.029	0.64–1.61	0.992	4 (16.0)	1.097	0.31–3.07	0.904	2 (14.3)	0.96	0.14–3.86	0.748
*T*. *trichiura*												
Lig.+Mod.	25 (75.8)	1.310	0.76–2.22	0.375	1 (25.0)	0.381	0.02–2.09	0.529	2 (100)	1.55	0.23–6.31	0.906
Heavy	0 (0.0)	NA	-	-	0 (0.0)	NA	-	-	0 (0.0)	NA	-	-
*A*. *lumbricoides*												
Lig.+Mod.	11 (33.3)	0.713	0.33–1.42	0.440	3 (75.0)	2.13	0.48–6.83	0.427	0 (0.0)	NA	-	-
Heavy	0 (0.0)	NA	-	-	1 (25.0)	0.65	0.03–3.63	0.988	0 (0.0)	NA	-	-
Hookworm												
Lig.+Mod.	0 (0.0)	NA	-	-	0 (0.0)	NA	-	-	0 (0.0)	NA	-	-
Heavy	0 (0.0)	NA	-	-	0 (0.0)	NA	-	-	0 (0.0)	NA	-	-

Nutritional status distribution (%) in STH infected schoolchildren of Nemba I (Gakenke district, Northern Province, Rwanda). N = number of schoolchildren of each group; n = number of schoolchildren parasitized; Lig.+Mod. = Light + Moderate infections; OR = Odd ratios; 95%CI = 95% confidence interval; NA = not available.

No STH infection intensity was significantly associated with anemia or with nutritional status (Tables 5 and 6).

## Discussion

The spectrum of parasite species found in this study is made up of 17 species, thus representing the broadest parasite spectrum ever found in Rwanda. A prevalence of 95.3% of schoolchildren were parasitized and 83.8% presented multiparasitism. Among protozoa, the different recognized symptoms-inducing and/or pathogenic parasites such as *Entamoeba* complex and *G*. *intestinalis* stand out, with the most prevalent protozoa being *Blastocystis* sp., actually related with nutritional problems like low weight and low height [32]. The fact that protozoa prevalence is above helminth prevalence is particularly striking, and could be related to the improvement of the living conditions of Nemba schoolchildren, especially after the implementation of a Neglected Diseases Control Program in which massive anthelminthic treatment is administered every two years in schoolchildren [19].

Among helminths, STHs stand out, presenting distinct geographical differences in prevalence in Rwanda [33, 34]. In the present study, *T*. *trichiura* was most prevalent, followed by *A*. *lumbricoides*, both with lower prevalence when compared to a previous study carried out in two other districts of the Northern Province (Musanze and Burera) [17] or even in different districts of the Southern Province, like Huye district [22, 35–38]. The prevalence of Hookworm was also low as in previous studies, except in the geo-statistical meta-analysis study [39], which found Hookworm to be the most prevalent helminth species.

No significant differences were found by gender for any parasite species detected, which could be explained by the lack of gender role differences. However, schoolchildren from 6 to 10 years of age were more affected by protozoa than helminth infections, with significant differences, likely to be due to the fact that their immune systems are still developing, and the risk of infection by any protozoa is greater as a result of common unhygienic behaviour. *A*. *lumbricoides* was the only one presenting a low percentage of heavy intensity infection, being in contrast with a national survey carried out in 2008, which involved over 8000 schoolchildren sampled from 30 districts, with the outstanding result of 66% of individuals infected by STHs [37], placing Rwanda among the most heavily infected STH endemic regions in the world [40].

Anemia is a medical condition of great impact, especially in developing countries. The prevalence of anemia in this study is similar to the one previously obtained in Northern Province [17]. Anemic schoolchildren did not present any significant difference by gender or age group, in contrast with Mupfasoni [17], who found 5-7-year old boys to be more likely to be anemic.

No direct relationship between anemia and intestinal parasites was detected, just as in previous studies carried out in Rwanda [17, 41, 42], but in contrast with Staudacher [37] who observed a relationship between anemia and geohelminths, and Heimer [38] who found *G*. *intestinalis* infection to be associated with low levels of hemoglobin, reaching 21.7% in anemic schoolchildren of Rwanda. Nonetheless, in other sub-Saharan African countries, a significant association between infected schoolchildren and the presence of anemia was detected [43].

Robertson [44] established that a low intensity multiparasite infection profile increased the odds of being anemic. However, in the present work we found that only 26.9% of schoolchildren infected with STH presented anemia, in accordance with the previous work [17] in which none of the concurrent multiparasite infections was significantly associated with anemia, likely to be due to the decrease of malaria.

In many populations, intestinal parasites are a source of nutritional stress affecting child growth. Our results on stunting agree with previous Rwandan studies in the Northern Province [17, 41] and are also similar to those from other sub-Saharan African countries [45]. Recently, chronic intestinal protozoa infections have become increasingly recognized as a cause of malnutrition in children [4]. The presence of *G*. *intestinalis* has been related to stunting in Rwanda [38] and the nutritional stress caused by this parasite may be related to the damage of the intestinal walls, leading to the malabsorption of nutrients.

We found males and older schoolchildren, to be most significantly affected by stunting, as supported by previous studies undertaken in that country [17, 38, 41]. This finding might be related to the fact that schoolchildren in advanced age may have a higher nutritional demand to reach adulthood, creating a greater need for energy than in younger schoolchildren. However, in other sub-Saharan African countries stunting was comparable between males and females and between schoolchildren of different age groups [4, 45].

Multiparasitism is also associated with stunting. Thus, children with low intensity infections of *T*. *trichiura* and *A*. *lumbricoides*—or when the first was of low intensity and the latter was found to be of low, moderate or heavy intensity–presented a higher risk of stunting. Likewise, Mupfasoni [17] found that Rwandan schoolchildren with coinfection, in moderate or heavy infections, suffered a higher risk of undernutrition.

Our study has some limitations: the low sensitivity of the Kato-Katz method in case of very low-intensity infections is noteworthy; the application of the method based on a single sample may impact prevalence measurements, above all, if eggs are highly clustered or in low abundance; and the need to process stool samples quickly after collection, especially for Hookworm eggs; the small sample size reduces the precision and weakens the statistical coefficients (ORs) making the use of multivariate logistic regression models impossible. Finally, no study of any kind was carried out on the existence of malaria among the students analyzed, although malaria is known not to be frequent in the Northern Province.

## Conclusions

The marked differences between parasite groups, with a high prevalence of protozoan species, underline the positive effect of the helminthic deworming campaigns carried out by the Rwandan government. The prevalence of anemia in Rwanda schoolchildren, at least in the Northern Province, is not a serious public health concern, but multiparasitism remains high among schoolchildren. Consequently, the priorities of reducing the prevalence of parasite infection in Rwandan schoolchildren should be centered on keeping up the anthelmintic plans initiated together with increased health education programs. Future studies, avoiding the limitations of the current one, would be convenient.

## Supporting information

S1 Table Anemia by gender and age-groups. Anemia distribution (%) by gender and age-groups among schoolchildren of Nemba I (Gakenke district, Northern Province, Rwanda), according to WHO (2001). N = number of schoolchildren studied; n = number of schoolchildren with anemia; OR = Odd ratios; 95%CI = 95% confidence interval.
(DOCX)

S1 File (XLS)
