# Supplementary material for: Anemia and undernutrition in intestinally parasitized schoolchildren from Gakenke district, Northern Province of Rwanda
Source: PLoS One. 2022 Jan 6;17(1):e0262361. doi: 10.1371/journal.pone.0262361 (PMC8735607; doi:10.1371/journal.pone.0262361)
Supplement: S1 Table — Anemia distribution (%) by gender and age-groups among schoolchildren of Nemba I (Gakenke district, Northern Province, Rwanda), according to WHO (2001). N = number of schoolchildren studied; n = number of schoolchildren with anemia; OR = Odd ratios; 95%CI = 95% confidence interval. (DOCX) [file pone.0262361.s001.docx]

**Supporting information**

**S1 Table**. **Anemia by gender and age-groups.**

|  |  |  | Anemia  N = 656 |  |
| --- | --- | --- | --- | --- |
|  |  | n (%) | OR (95%CI) | *P* value |
| Gender |  |  |  |  |
|  | Male | 11 (42.3) | 0.729 (0.32-1.61) | 0.552 |
|  | Female | 15 (57.7) |  |  |
| Age-groups (years) |  |  |  |  |
|  | 5-12 | 16 (61.5) |  |  |
|  | 13-15 | 10 (38.5) | 1.614 (0.72-3.72) | 0.322 |
|  | >15 | 0 (0.0) |  |  |
| Total |  | 26 (4.0) |  |  |

Anemia distribution (%) by gender and age-groups among schoolchildren of Nemba I (Gakenke district, Northern Province, Rwanda), according to WHO (2001). N= number of schoolchildren studied; n= number of schoolchildren with anemia; OR= Odd ratios; 95%CI= 95% confidence interval.
